# Development and validation of a mutation-based model to predict immunotherapeutic efficacy in NSCLC

**DOI:** 10.3389/fonc.2023.1089179

**Published:** 2023-02-24

**Authors:** Ping He, Jie Liu, Qingyuan Xu, Huaijun Ma, Beifang Niu, Gang Huang, Wei Wu

**Affiliations:** ^1^ Department of Cardiac Surgery, Southwest Hospital, Army Medical University (Third Military Medical University), Chongqing, China; ^2^ Department of Thoracic Surgery, Southwest Hospital, Army Medical University (Third Military Medical University), Chongqing, China; ^3^ Computer Network Information Center, Chinese Academy of Sciences, Beijing, China; ^4^ University of the Chinese Academy of Sciences, Beijing, China; ^5^ Department of Biochemistry and Molecular Biology, College of Basic Medical Science, Army Medical University (Third Military Medical University), Chongqing, China

**Keywords:** non-small cell lung cancer, immunotherapy, mutation-based model, immune features, biomarker

## Abstract

**Background:**

Immunotherapy has become increasingly important in the perioperative period of non-small-cell lung cancer (NSCLC). In this study, we intended to develop a mutation-based model to predict the therapeutic effificacy of immune checkpoint inhibitors (ICIs) in patients with NSCLC.

**Methods:**

Random Forest (RF) classifiers were generated to identify tumor gene mutated features associated with immunotherapy outcomes. Then the best classifier with the highest accuracy served for the development of the predictive model. The correlations of some reported biomarkers with the model were analyzed, such as TMB, PD-(L)1, KEAP1-driven co-mutations, and immune subtypes. The training cohort and validation cohorts performed survival analyses to estimate the predictive efficiency independently.

**Results:**

An 18-gene set was selected using random forest (RF) classififiers. A predictive model was developed based on the number of mutant genes among the candidate genes, and patients were divided into the MT group (mutant gene ≥ 2) and WT group (mutant gene < 2). The MT group (N = 54) had better overall survival (OS) compared to the WT group (N = 290); the median OS was not reached vs. nine months (P < 0.0001, AUC = 0.73). The robust predictive performance was confifirmed in three validation cohorts, with an AUC of 0.70, 0.57, and 0.64 (P < 0.05). The MT group was characterized by high tumor neoantigen burden (TNB), increased immune infifiltration cells such as CD8 T and macrophage cells, and upregulated immune checkpoint molecules, suggesting potential biological advantages in ICIs therapy.

**Conclusions:**

The predictive model could precisely predict the immunotherapeutic efficacy in NSCLC based on the mutant genes within the model. Furthermore, some immune-related features and cell expression could support robust efficiency.

## 1 Introduction

With the emergence of immune checkpoint inhibitors (ICIs), such as programmed cell death protein-1 (PD-1)/programmed cell death protein-1 ligand (PD-L1) and cytotoxic T lymphocyte-associated 4 (CTLA-4) inhibitors, immunotherapy has become one of the most promising treatment strategies for lung cancer, particularly for advanced and metastatic non-small cell lung cancers (NSCLC) with no epidermal growth factor receptor (*EGFR*) or anaplastic large cell kinase gene (*ALK*) alterations (NCCN Guidelines Version 3.2022 Non-Small Cell Lung Cancer, https://www.nccn.org/). Recently, the amazing breakthrough of Checkmate-816 (ClinicalTrials.gov number, NCT02998528) phase 3 results showed that neoadjuvant nivolumab plus chemotherapy resulted in significantly longer event-free survival and a higher percentage of patients with a pathological complete response (pCR) than chemotherapy alone (24% vs. 2.4%) (1). Immunotherapy has become more important in the perioperative period of NSCLC.

Since the FDA approved pembrolizumab for treating tumor mutational burden-high (TMB-H) patients, TMB became an indication for cancer therapy for the first time (2). Subsequently, PD-(L)1 expression, TMB level (TMB-H/TMB-L), and microsatellite instability/defective mismatch repair (MSI/dMMR) status helped screen patients for enduring benefits of ICI therapy. However, the tumor immune microenvironment presents complex and distinct characteristics in various tumor types. Gu et al. identified that microenvironments respond differently to ICIs in different tumors derived from the same cancer cells (3). Accordingly, a more informative predictive model that integrates diversity biomarkers associated with immunotherapy outcomes and resistance in NSCLC is urgently required (4).

Advances in gene sequencing technology, bioinformatics, and deep learning analysis methods have considerably facilitated the discovery of clinically meaningful genetic signatures. Furthermore, with the increasingly abundant tumor sequencing data published in the literatures and The Cancer Genome Atlas (TCGA) database, studies in cancer prognosis and therapy-related biomarkers have been conducted to address crucial clinical questions. Sinha et al. identified that M1 macrophages and dendritic cells were strongly correlated with high TMB, which could stratify the immunotherapy responders well (5). Chen et al. identified that the apolipoprotein B mRNA editing enzyme, catalytic polypeptide-like (APOBEC) signature was significantly associated with ICIs therapeutic efficacy in NSCLC (6). Zhang et al. analyzed the correlation of single-gene mutation events with PFS data after ICI therapy, and the mutations that occurred in serine/threonine kinase 11 (*STK11*) were found to be related to poorer PFS. In contrast, the mutated protein tyrosine phosphatase receptor type D (*PTPRD*) was associated with better PFS (7). On this basis, we confirmed that gene mutations might exhibit distinct and complex associations with immunotherapy response. Zhang et al. emphasized the interaction effects of co-mutations on ICIs efficacy and constructed an inter-model as a predictor for ICI therapy (7). In summary, genetic alterations in tumor cells are always caused by DNA damage, pathway disorder, and heterogeneous immune tumor microenvironment (TME). The accumulated abnormal somatic mutations could affect tumor susceptibility to ICIs treatment (8).

We used the random forest classifier to screen the superior gene feature set, which considered the multiple gene accumulation effects in tumor cells. Finally, a mutation-based model was proposed to predict the therapeutic efficacy of ICIs in NSCLC, especially for lung adenocarcinoma (LUAD) and lung squamous cell carcinoma (LUSC) patients. The advantage of this tool is that the tumor immunogenic neoantigen generated from tumor somatic mutations is more specific for patients with diverse NSCLCs.

## 2 Results

### 2.1 Development of a mutation-based model to predict immunotherapy response for NSCLC

**Figure 1:**
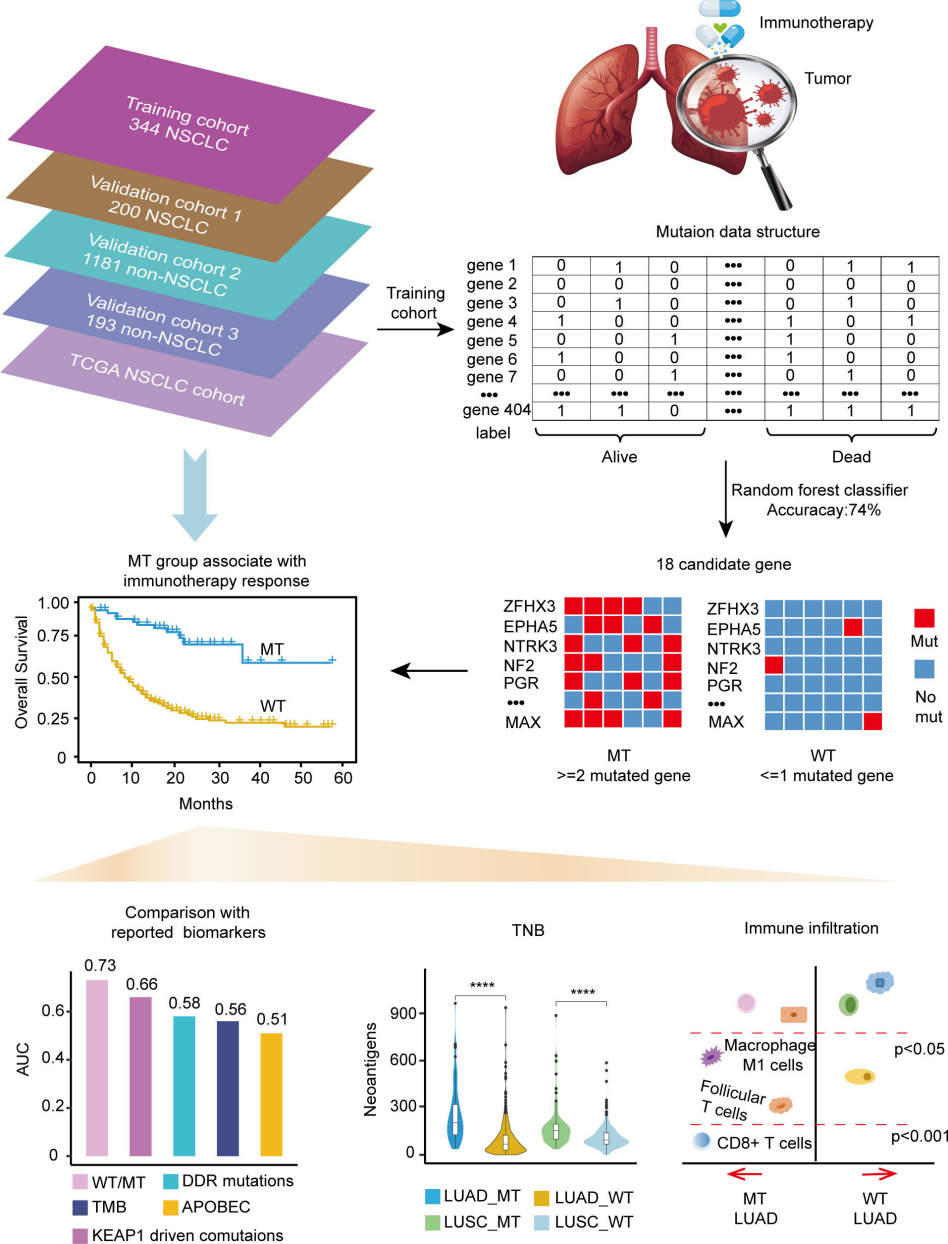
Workflow of the study.

The training cohort’s somatic mutational profiles of 344 patients with NSCLC were analyzed and sequenced using an MSK-IMPACT target sequencing panel (9). The mutational landscape showed that the top five frequently mutated genes were tumor protein p53 (*TP53*), Kirsten rat sarcoma viral oncogene homolog (*KRAS*), kelch like ECH associated protein 1 (*KEAP1*), *STK11*, and *PTPRD* (**Supplementary Figure 1**). Thus, we aimed to develop a mutation-based model to predict immunotherapeutic efficacy in NSCLC, and the detailed workflow is shown in **Figure 1**.

**Figure 2:**
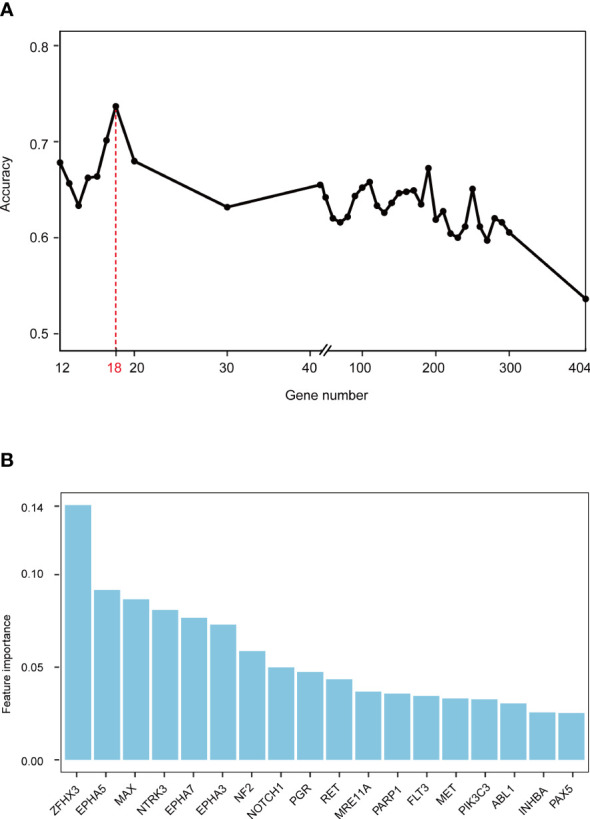
Screening feature genes using random forest (RF) algorithm. **(A)** Feature combination selection. The combination of 18 genes can achieve the highest classification accuracy with the least number of variables. **(B)** Feature importance of 18 genes.

Gene mutations and survival status in the training cohort were converted to binary variables in advance. The process and result of feature selection are shown in **Figure 2A**. With the deletion of unimportant features, the overall classification accuracy tended to increase, mainly because eliminating irrelevant and redundant features improved classifier performance. When the classification accuracy reached the highest value, it began to show a downward trend. The combination of 18 genes (**Table 1**), such as zinc finger homeobox 3 (*ZFHX3*) and EPH receptor A5 (*EPHA5*), achieved the highest classification accuracy with the least number of variables, and the accuracy rate reached 73.68% (**Figure 2A**). These 18 genes thus served as candidate genes, and the importance score of each gene is displayed in **Figure 2B**.

**Table 1:** List of 18 genes whose mutations could predict immunotherapy outcomes in NSCLC.

Gene Name (% with mutations in the training cohort)			
*ZFHX3* (8%)	*NTRK3* (6%)	*EPHA7* (5%)	*EPHA5* (8%)
*NF2* (3%)	*ABL1* (2%)	*MAX* (2%)	*PARP1* (1%)
*PAX5* (1%)	*PGR* (4%)	*FLT3* (3%)	*MRE11A* (2%)
*PIK3C3* (4%)	*INHBA* (3%)	*RET* (3%)	*EPHA3* (11%)
*MET* (4%)	*NOTCH1* (4%)		

**Figure 3:**
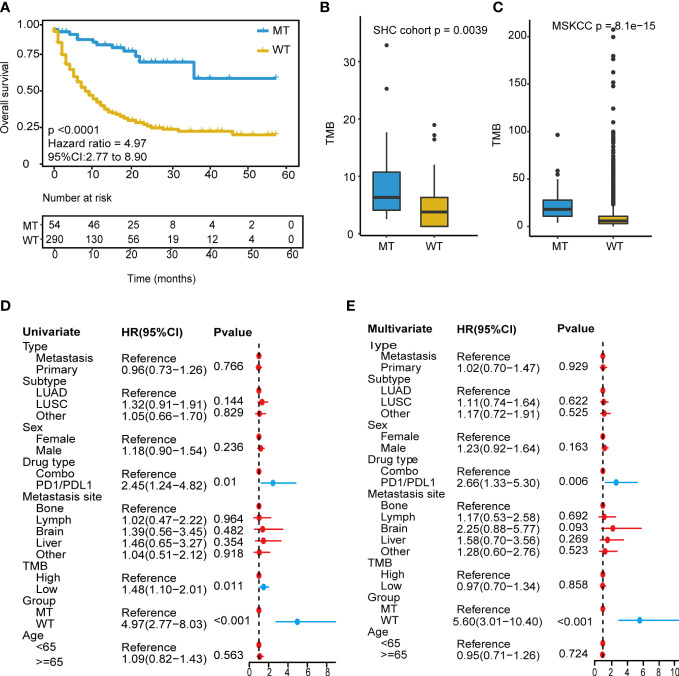
Development of the mutation-based model. **(A)** Kaplan-Meier survival analysis for OS between MT group and WT group in the training cohort. **(B, C)** TMB analysis between MT group and WT group in SHC cohort and training cohort. **(D)** Univariate Cox regression analysis of clinical factors and the model for prognosis after ICIs treatment. **(E)** Multivariate Cox regression analysis of clinical characteristics and the model for prediction after ICIs treatment. OS, overall survival; MT group, patients with ≥2 mutant genes; WT group, patients with <2 mutant gene; TMB-H, tumor mutational burden-high (≥10 mutation/Mb); TMB-L, tumor mutational burden-low (<10 mutation/Mb); ICIs, immune checkpoint inhibitors.

We focused on mutation frequency, count, and the corresponding sample number of the 18 genes. Firstly, we compared the predictive ability of the wt group (mutant gene=0), single mutant group (mutant gene=1), two mutant group (mutant gene =2), three mutant group (mutant gene=3), etc., until seven mutant group (mutant gene=7) according to the mutation status of 18 candidate genes (**Supplementary Figure 2A**). The median OS was 8, 13, and 36 months for wt, Single mutant, and 2 mutant group respectively, and the 2 mutant group has an elevated median OS than the other 2 groups. When the mutant gene>2, the OS was not reached, and the patients’ number dropped significantly. Then, these results revealed the superior OS advantage of the patients harboring at least 2 mutant candidate genes when compared with wt or single mutant group. Thus, the mutation-based predictive model defined mutant gene≥ 2 as MT group, which could benefit more from ICIs therapy. Afterward, a mutation-based model was determined to discriminate the differential immunotherapy outcomes in the training cohort. According to the mutation status of 18 genes, patients with ≥ 2 mutant genes were classified into the MT group, while those with < 2 mutant genes were in the WT group. The survival analysis showed that the MT group had better OS (N = 54, median OS not reached) than the WT group (N = 290, median OS 9 months, *P* < 0.0001, hazard ratio (HR) = 4.97, 95% CI [2.77,8.90]) (**Figure 3A**), and the AUC value was 0.73 (**Supplementary Figure 2B**). Then, we explored the mutation frequency and distribution of these 18 genes in MT group of the training cohort. The mutation landscape was shown in **Supplementary Figure 2C**, which seems dispersed for mutant genes within samples. Moreover, we analyzed the overlapping genetic alteration of 18 genes within patients in the MT group (**Supplementary Table 1**), and an almost 50% overlapping rate was seen with no regular combination pattern. We don’t find the typical distribution of genetic alteration of these 18 genes in the MT group, which further prove the predicting ability of the 18-gene model is the accumulative effect of 18 genes rather than the contribution of part genes. All the results demonstrated the accurate predicting performance of our model.

Owing to excellent performance, we further demonstrated the independent predictive power of the model. Univariate Cox regression analysis revealed that the drug (PD-1/L1 vs. combination strategy), TMB level, and our model had a significant correlation with the hazard ratio (all *P* < 0.05) (**Figure 3D**). In contrast, the TMB level showed weak performance in the multivariate Cox regression analysis (**Figure 3E**). Our model consistently had the lowest *P* value < 0.001.

Next, a comparative analysis was conducted between the model and the TMB level. TMB_H group had significantly longer OS (TMB ≥ 10 mutation/Mb, N = 112, P = 0.0099, median OS 18 months) than the TMB_L group (TMB < 10 mutation/Mb, N = 232, P = 0.0099, median OS 10 months) (**Supplementary Figure 3A**), and the AUC value was 0.56 (**Supplementary Figure 3B**). The combined analysis revealed that the long-term survival benefits from immunotherapy were significantly superior in the MT group compared to that in the WT group, regardless of TMB level (**Figure 3C**). The model based on 18-gene mutation status was superior to the TMB level in predicting the therapeutic efficacy of ICIs.

Furthermore, correlation analysis with TMB was conducted, and the MT group showed a significantly high TMB than those in the WT group (P<0.001) (**Figure 3C**). The non-therapy samples from the southwest hospital clinical (SHC) cohort were also divided into MT (14/82) and WT (68/82) groups based on the mutation-based model, and the TMB of the MT group remained higher than those of the WT group (P=0.0039) (**Figure 3B**). In addition, all patients were microsatellite-stable (MSS), and there were no differences shown in HLA variability between the MT and WT groups (**Supplementary Figure 3C**). Although the lack of therapy and survival prognosis information, the SHC cohort could reveal the real-world mutation landscape for NSCLC in the Chinese (**Supplementary Figure 4**).

### 2.2 Association analysis between the mutation-based model and other predictive biomarkers of NSCLC

Recently, an increasing number of biomarkers have been identified to predict the therapeutic efficacy of ICIs in NSCLC, including *KEAP1*-driven co-mutation events, DDR (DNA damage repair) pathway, APOBEC signature, and others (10–13). We performed the following analyses to verify whether our model outperformed other predictors and the correlation with these biomarkers. The mutation data of 344 NSCLC patients from the training cohort was used for the following correlation analysis.

**Figure 4:**
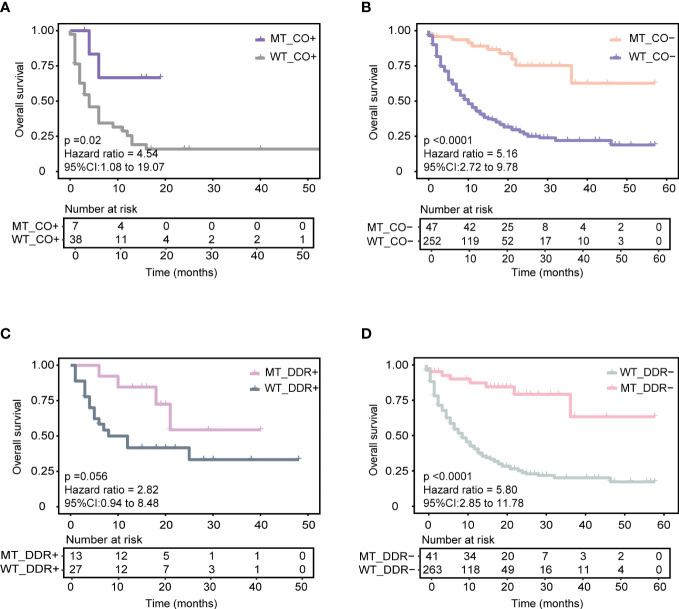
Association of our model with other reported biomarkers in predicting ICIs responses based on the training cohort. **(A)** Predicting the performance of the model in patients with *KEAP1-*driven co-mutation subgroup (CO+) in the training cohort. **(B)** Predicting the performance of the model in patients without *KEAP1-*driven co-mutation subgroup (CO-) in the training cohort. **(C)** Predicting the performance of the model in patients with more than or equal to one of five DDR gene mutations (DDR+) in the training cohort. **(D)** Predicting the performance of the model in patients with none of five DDR gene mutations subgroup (DDR-) in the training cohort.

First, *KEAP1*-driven co-mutations were reported to be correlated with immunotherapy response in LUAD patients. Co-mutation in at least two genes among *KEAP1*, *STK11*, polybromo-1 (*PBRM1*), and SWI/SNF-related, matrix-associated, actin-dependent regulator of chromatin, subfamily A, member 4 (*SMARCA4*) was related to a lack of response to immunotherapy, despite high TMB (14). The training cohort was used for comparison, and the patients with *KEAP1-*driven co-mutation (CO+) (N = 45) were found to have a worse prognosis than those without *KEAP1-*driven co-mutation (CO-) (N = 299) (median OS 6 months vs. 13 months, P = 0.0091, HR = 1.656, 95% CI [1.14,2.41]) (**Supplementary Figure 5A**). The AUC value was 0.66 (**Supplementary Figure 5B**). Then, the CO+ and CO- groups were compared with our model separately. For CO+ patients, the MT_CO+ subgroup (N = 7) had longer OS than the WT_CO+ subgroup (N = 38) (median OS not reached vs. four months, P = 0.02, HR = 4.54, 95% CI [1.08,19.07]) (**Figure 4A**). Combined with our model, seven patients achieved good OS despite *KEAP1-*driven co-mutation. For CO- patients, the MT_CO- subgroup (N = 47) had significantly better OS than the WT_CO- subgroup (N = 252) (*P* < 0.0001, HR = 5.16, 95% CI [2.72,9.78]) (**Figure 4B**).

Next, we identified the correlation of the model with a five-gene signature of the DDR pathway, which has been reported to positively correlate with ICI therapy benefit. Five candidate genes of the DDR pathway, including mutS homolog 2 (*MSH2*), mutS homolog 6 (*MSH6*), PMS1 homolog 2 (*PMS2*), Polymerase-epsilon (*POLE*), and breast cancer 2 early onset (*BRAC2*) genes, were obtained from Conway et al. (10). In the training cohort, patients with ≥ 1 mutant DDR gene had longer OS (DDR+) (N = 40) than those with no mutant DDR gene (DDR-) (N = 304) (median OS 21 months vs. 11 months, P = 0.091, HR = 1.48, 95% CI [0.93,2.35]) (**Supplementary Figure 5C**). The AUC value was 0.58 (**Supplementary Figure 5D**). Similarly, the DDR+ and DDR- groups were compared with our model separately. Combined with our model, the MT_DDR+ subgroup (N = 13) had superior OS benefits than the WT_DDR+ subgroup (N = 27) (median OS not reached vs. ten months, P = 0.056, HR = 2.82, 95% CI [0.94,8.48]) (**Figure 4C**). For DDR- patients, the MT_DDR- subgroup (N=41) had better OS than the WT_DDR- subgroup (N = 263) (median OS not reached vs. nine months, *P* < 0.0001, HR = 5.80, 95% CI [2.85,11.78]) (**Figure 4D**). Compared with our model, 41 patients with DDR- but in the MT group also had better survival (**Figure 4D**); such results could help improve clinical therapeutic decisions.

Lastly, we explored the correlation of the APOBEC gene signature with our model. APOBEC-related mutations were significantly increased owing to the increased expression of the *APOBEC3B* gene, and patients with upregulated *APOBEC3B* treated with ICIs had a poor prognosis (13). In the training cohort, patients with ≥ 1 APOBEC-related gene mutation (APOBEC+) did not have significant OS benefits compared to those with no APOBEC-related gene mutations (APOBEC-) (P = 0.67) (**Supplementary Figure 6A**). The AUC value was 0.51 (**Supplementary Figure 6C**). Combined with our model, patients in the MT group had a significantly better prognosis than those in the WT group, regardless of the APOBEC gene mutational status (**Supplementary Figure 6B**).

These biomarkers thus showed enhanced predictive performance when combined with our model, which could help more patients attempt immunotherapy. These results indicate that more attempts are needed from various perspectives to develop more powerful predictive biomarkers.

### 2.3 Validation of the mutation-based model

**Figure 5:**
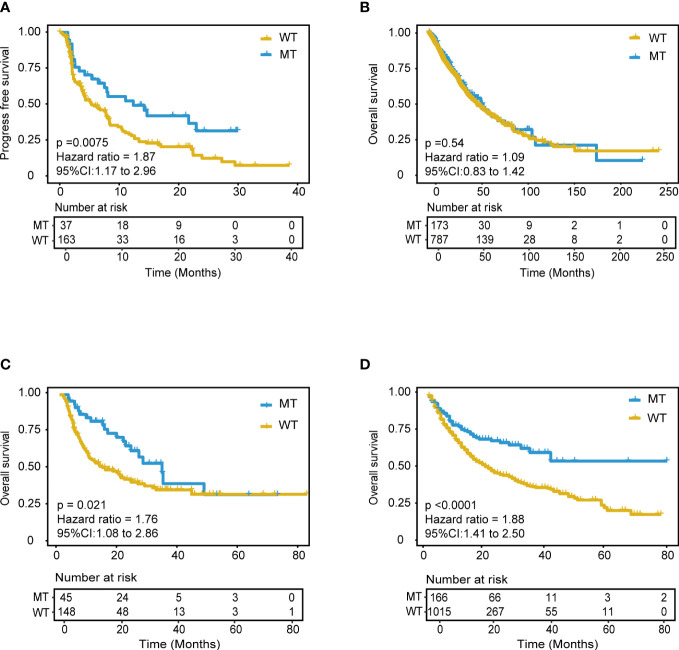
Validation of the mutation-based model in validation cohorts. **(A)** Kaplan-Meier survival analysis for PFS between MT group and WT group in the validation cohort 1. **(B)** Kaplan-Meier survival analysis for OS between MT group and WT group in the non-immunotherapy TCGA cohorts. Extension validation of the model in the two independent pan-cancer cohorts. **(C)** Validation cohort 2 and **(D)** Validation cohort 3. OS, overall survival; PFS, progression-free survival.

With the same grouping criteria, validation cohort 1, integrated from three public NSCLC WES (whole-exome sequencing) cohorts, including 56 patients with NSCLC treated with anti-PD-(L)1 therapy (15), 75 patients with NSCLC treated with PD-1 plus CTLA-4 blockade (LUAD only) (16), and 69 patients with NSCLC treated with anti-PD-(L)1 monotherapy at Sun Yat-sen University Cancer Center (SYSUCC) (17), included 200 ICI-treated patients and was used to validate the predictive ability of the model. The results showed superior PFS in the MT group (median PFS 12.4 months) compared with that in the WT group (P = 0.0075; HR = 1.87, 95% CI [1.17,2.96], median PFS 5.4 months) (**Figure 5A**), and the AUC value was 0.70 (**Supplementary Figure 7H**). Moreover, the subgroups analysis of wt, single, and compound mutant (mutant gene ≥ 2) group was also conducted in the validation cohort 1, and the median PFS was 4.3, 8.4, and 12.4 months respectively. The validation data further confirmed the immunotherapeutic advantage of the NSCLC patients with ≥ 2 mutant model genes.

Additionally, we performed the performance comparison analysis between two representative NSCLC subtypes in the training cohort and validation cohort 1. 41 (13.23%) LUAD and 6 LUSC (1.94%) patients fall in the MT group in the training cohort (**Supplementary Figure 7A**). For LUAD subtype (N=266), patients in the MT group had better OS than those in the WT group (P<0.0001). For LUSC subtype (N=44), patients in the MT group had better OS than those in the WT group (P<0.0001) (**Supplementary Figure 7B**). The subtype analysis further confirmed the robustness of the model for predicting immunotherapy efficiency in the NSCLC. To validate the robustness of the mutation-based predicting model, the same comparison analysis was performed in validation cohort 1. There were 32 LUAD (16.67%) and 3 LUSC (1.56%) patients classified into the MT group in the validation cohort 1 (**Supplementary Figure 7C**). Similar to the training cohort, the LUAD patients in MT group (N=32) benefit more PFS from ICIs therapy than those in the WT group (N=132) (P=0.066). For LUSC subtype (N=28), patients in the MT group (N=3) had longer PFS than those in the WT group (N=25) (**Supplementary Figure 7D**). According to the above results, the proportion of MT and WT group are similar in the LUADs and LUSCs, which reveals that our mutation-based model is stable and independent of NSCLC subtypes. These results indicated the stability and robustness of the model in predicting immunotherapy efficiency for the different NSCLC subtypes.

Furthermore, we also performed a comparative analysis between the mutation-based model and TMB, *KEAP1*-driven co-mutation, DDR (DNA damage repair) pathway signature in the validation cohort 1, and the corresponding survival curve and AUC are shown in **Supplementary Figures 7E–H**. The AUC of TMB, *KEAP1*-driven co-mutation, and DDR were 0.64, 0.54, and 0.51, respectively, which were all lower than the AUC of the mutation-based model (AUC=0.7). Finally, the mutation-based model consistently performed better in predicting the immunotherapeutic efficiency of the NSCLC patients in the validation cohort 1. As our model showed a sound and robust predicting power both in the training cohort and validation cohort 1, we confirmed that it could independently predict response to immunotherapy.

Furthermore, we hypothesized that the predictive power was only specific to immunotherapy response in NSCLC rather than other tumor-related prognoses. Further analyses were explored using the mutation and mRNA data of non-ICI treatment TCGA NSCLC patients, including the mutation profile of LUADs and LUSCs. The results of mutation data analysis showed no significant PFS difference between the MT and WT groups (P = 1) (**Figure 5B**). Furthermore, the RNA-seq data were used to explore the RNA expression differences of the 18 genes in TCGA NSCLC patients. There was no difference between the MT and WT groups (P = 0.54) (**Supplementary Figure 8**).

The above analyses indicated that our model had specific predictive power for the immunotherapy response of patients with NSCLC rather than for survival prognosis.

### 2.4 Robust analysis of the mutation-based model across pan-cancer cohorts

Extensive validation was conducted using the mutation data of two pan-cancer cohorts. Validation cohort 2 included 1181 patients treated with anti-PD-(L)1 therapy (From Samstein cohort, 350 patients with NSCLC, and 130 patients with unknown cancer were excluded) (9). Validation cohort 3 included 193 patients treated with anti-PD-(L)1 therapy (15) (From Miao cohort, 56 patients with NSCLC were excluded). Remarkably, the model still showed good predictive power in validation cohort 2 (*P* < 0.0001, HR = 1.88, 95% CI [1.41,2.50]) (**Figure 5D**). Patients in the MT group could achieve better outcomes with ICI treatment within approximately four years. This predictive performance was also identified in validation cohort 3 (P = 0.021, HR = 0.76, 95% CI [1.08,2.86]) (**Figure 5C**). The AUC value in validation cohort 2 and 3 was 0.57 and 0.64, respectively. These results suggested the model’s robust and universal predictive ability across pan-cancer, which deserves further exploration in different tumor types.

### 2.5 Immune-related features of the MT group and WT group

**Figure 6:**
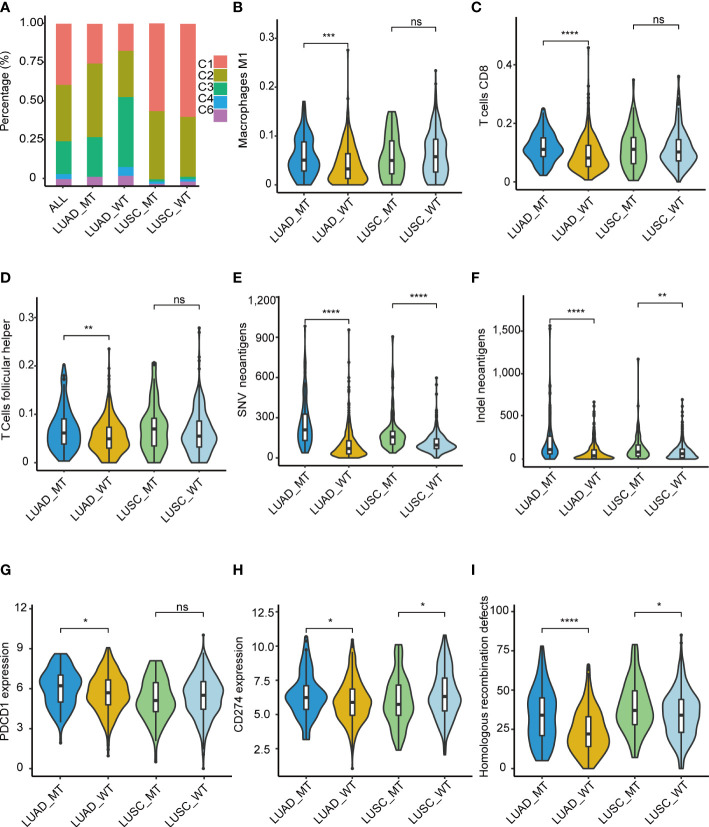
Immune-related features of the model in the TCGA cohorts. **(A)** Distribution difference of five immune subtypes in the TCGA cohorts. **(B)** M1 macrophage, **(C)** T.cells.CD8, **(D)** T.cells.follicular.helper. Comparison analysis of Comparison analysis of TNB among four groups of LUAD_MT, LUAD_WT, LUSC_MT, and LUSC_WT. **(E)** SNV, **(F)** InDel. Immune infiltration analysis revealed four significantly different cells according to the RNA gene expression data of TCGA among four groups of LUAD_MT, LUAD_WT, LUSC_MT, and LUSC_WT. **(G)** *PDCD1*(PD-1) gene and **(H)** *CD274*(PD-L1) gene expression among four groups of LUAD_MT, LUAD_WT, LUSC_MT, and LUSC_WT. **(I)** Comparison analysis of HRD gene signature score among four groups of LUAD_MT, LUAD_WT, LUSC_MT, and LUSC_WT. *P ≤ 0.05; **P ≤ 0.01; ***P ≤ 0.001; ****P ≤ 0.001; ns, not significant. LUAD, lung adenocarcinoma; LUSC, lung squamous cell carcinoma; SNV, single nucleotide variant; InDel, insertion-deletion. *PDCD1* gene, coding gene of PD-1 protein; *CD274*, coding gene of PD-L1 protein; HRD, Homologous recombination defects.

Immune-related features analysis was conducted using the mutation data and mRNA expression data of TCGA-LUAD and TCGA-LUSC, and grouping criteria for immune subtypes, immune cell fraction, etc., were referring to the method of original literature. Thorsson et al. identified six immune subtypes (C1: wound healing, C2: IFN-γ dominant, C3: inflammatory, C4: lymphocyte depleted, C5: immunologically quiet, and C6: TGF-β dominant), which defined immune response patterns impacting prognosis (18). Thus, we explored whether immune subtypes differed between the two groups (MT and WT). Patients in the MT group presented a higher percentage of C1 and C2 subtypes compared with those in the WT group, while C3, C4, and C6 subtypes were more enriched in the patients in the WT group (**Figure 6A**). Also, we found that LUSCs presented higher C1 and C2 than LUADs, which was consistent with the study of Thorsson et al. (18). Taken together, immune subtypes could further reveal the heterogeneous characteristics of tumors.

To further explore the immune-related features of our model in NSCLC, we compared the differential immune infiltrating cells in the immune TME between the MT group and the WT group. Dendritic resting cells, monocyte cells, memory B cells, mast cells, and Treg cells were lower in the MT group than in the WT group. In contrast, CD8 T cells, macrophages cells, plasma cells, and follicular T cells were enriched in the MT group (**Supplementary Figure 9**). Given the above differences in immune subtypes between LUAD and LUSC, differential immune infiltrating cells between LUADs and LUSCs were analyzed. M1 macrophage (**Figure 6B**), T. cells.CD8 (**Figure 6C**), and T.cells.follicular.helper (**Figure 6D**) were significantly more abundant in the MT LUADs (*P* < 0.01). UMAP dimensionality reduction was performed to show the distribution of the different cell types: T cells, macrophage, epithelial cells, B cells, iPS cells, tissue stem cells, and endothelial cells (**Supplementary Figure 10**). Furtherly, we analyzed the expression profiles of 18 genes in the above cell types. As the most critical gene in the RF analysis, *ZFHX3* is more often expressed in macrophage and epithelial cells. Other genes showed significant enrichment in T cells and macrophage cells, except for EPH receptor A3 (*EPHA3*), *EPHA5*, EPH receptor A7 (*EPHA7*), and progesterone receptor (*PGR*) genes, which were hardly expressed in the above cell types (**Supplementary Figure 11**).

In summary, immune cells exhibited heterogeneous immunogenicity in the TME. According to our model, CD8 T cells, macrophage cells, and helper T cells were the main force during the response to ICIs. McGrail et al. identified that CD8 T cell infiltration exhibited a significantly positive correlation with tumor neoantigen burden (TNB) in LUAD and an opposite pattern in LUSC (19). Next, we described the potential correlation between TNB and immunotherapy outcomes. TCGA mutation data (both SNV (single-nucleotide variant) and InDel (insertion-deletion) were used for the comparative analyses of the TNB (**Figures 6E, F**). Patients in the MT group had a significantly higher TNB in LUADs and LUSCs than in the WT group. LUADs had a considerably higher level of TNB than LUSCs.

In addition to high T cell infiltration and TNB, the MT group presented upregulated mRNA expression of PD-1(**Figure 6G**) and PD-L1 (**Figure 6H**) genes. The significant upregulation was only present in the LUADs; in the LUSCs, the pattern was opposite or non-significant. Despite this, the results further demonstrated the superior immunogenicity of the MT group. We also identified a correlation between interferon-gamma (IFN-γ), N6 -methyladenosine (m6A), and Homologous recombination deficiency (HRD) gene signatures with our model. Recently, Zhou et al. identified that homologous recombination (HR) gene deficiency was positively associated with an improved response to immuno-neoadjuvant treatment in NSCLC (12). Comparison analysis was conducted, and the HRD score was used to quantify defects in homologous recombination. The results showed that the MT group had higher HRD scores than the WT group for both LUADs and LUSCs (*P* < 0.01) (**Figure 6I**). The result indicated that patients with HRD were more likely to benefit from immunotherapy in NSCLC, which was consistent with the findings of previous studies (12).

*IFNγ* gene signature has been reported to be positively correlated with ICIs treatment outcomes due to the ability to activate tumor microenvironment cells such as T cells and Natural killer (NK) cells (20). The results revealed a significantly higher *IFNγ* gene signature score in the MT group, specifically in the LUADs (**Supplementary Figure 12A**). As for m6A genes, Li et al. systematically reviewed that m6A modification could affect the immune response in the TME. Some of the m6A eraser, writer, and reader genes were positively correlated with the ICIs treatment outcomes (21). Our results showed that m6A eraser genes had lower expression in the MT group than in the WT group, while m6A writer and reader genes had higher expression in the MT group (**Supplementary Figures 12B–D**). Interestingly, the findings showed an opposite or insignificant correlation in LUSCs, which needs further confirmation.

Due to the immune advantage in LUADs, we further performed the pathway enrichment analysis using RNAseq data of the LUADs. The unsupervised clustering heatmap between the MT and WT groups is shown in **Supplementary Figure 13**.

## 3 Discussion

Biomarker-guided immunotherapy applications warrant precise stratification of patients using sensitive and specific biomarkers. NSCLCs with high TMB and TNB tend to benefit from immunotherapy (6). Furthermore, tumor somatic mutations can reveal tumor-specific significance, such as tumor progress or resistance, and indirect effects on the TME, such as immune-suppressive or immune-supportive effects on tumor cells. Herein, we developed a mutation-based model to predict immunotherapeutic efficacy based on long-term survival outcomes of patients with NSCLC treated with ICIs. The prognosis analysis showed highly significant differences between MT and WT groups in OS and PFS outcomes. Some studies have shown that mutations in *ZFHX3*/*EPHA4*/*EPHA7* genes are closely related to better response to ICIs in multiple cancers. These gene mutations were always accompanied by enhanced infiltration of CD8 T cells and M1 macrophages, increased TMB level and decreased immune-suppressive regulatory T cells (Tregs), consistent with our study (22–24). Interestingly, although not all of the mutant genes have prognostic significance for ICI treatment in NSCLC, the interactions of these 18 genes are meaningful for ICI therapy efficacy.

Notably, this is the first study to screen the best classifier capable of predicting immunotherapy outcomes using the RF algorithm. The 18-gene set with the least number of variables and the highest classification accuracy rate was used to develop the model. Then, comparing the predictive performance of different grouping criteria, patients who harbor at least 2 mutant candidate genes had the superior advantage when treated with ICIs, and the mutation-based model was established. Subsequently, a series of further explorations were carried out from different perspectives. Univariate and multivariate Cox regression analyses showed that the model had a superior predictive power over other factors, such as tumor type, sex, and TMB. Surprisingly, independent of the TMB level, the MT group consistently achieved better survival, which revealed the robust predictive power of our model. Furthermore, the superior predictive power of our model may explain the effectiveness of immunotherapy in TMB-L patients with NSCLC. Our model does not have cutoff value issues, unlike PD-(L)1 and TMB, resulting in the specific predictive advantage in NSCLC.

Furthermore, the mRNA expression analysis in the TCGA NSCLC cohort showed no significant difference in the expression of the 18 genes. Survival prognosis using the mutation data of TCGA NSCLC revealed no different between the MT and WT groups. The results indicated that our model had the specific predictive power for the immunotherapy response of patients with NSCLC rather than prognosis biomarkers. Our model demonstrated robust and universal predictive ability across pan-cancer, which deserves further exploration in different tumor types. The AUC value is higher in the training cohort and validation cohort 1 compared with validation cohorts 2 and 3, which may be because the mutation-based model is developed specifically for NSCLC patients. More profound pan-cancer cohorts are needed to optimize the immunotherapy predicting model. According to the analysis in our SHC cohort, a positive correlation of the mutation-based model with TMB also suggests reliable predictive ability in the Chinese NSCLC cohort.

A growing number of biomarkers are being studied to predict prognostic significance for immunotherapy in patients with NSCLC. *KEAP1*-driven co-mutation events, DDR pathway gene signature, and APOBEC gene signature were chosen as representative biomarkers for comparison analysis of predictive performance. When combined with our model, some patients who were initially regarded as immunotherapy non-responders could be reclassified as responders. These results implied synergistic effects of these immunotherapy-related biomarkers. The reported biomarkers and our model could complement each other as immunotherapy biomarkers, which indicates that more attempts are needed from various perspectives to develop more powerful predictive biomarkers. On the other hand, by comparing the predicting performance of TMB, *KEAP1*-driven co-mutation events, and DDR pathway gene signature, the mutation-based model revealed robust predictive ability and outperformed the AUC value both in the training cohort and validation cohort 1.

Moreover, the elevated signature scores of HRD genes, IFN-γ genes, and the m6A genes also denoted an immune-responsive feature of the MT LUADs. For LUSCs, the correlation seemed ambiguous. A series of differences may be caused by an unbalanced sample number of LUADs and LUSCs, and most NSCLC samples were LUAD in the cohorts. However, the detailed performance comparison between these two representative NSCLC subtypes in the training and validation cohort1 indicated the accurate and robust predictive ability of the mutation-based model for ICIs therapeutic efficiency in the NSCLC.

Crucially, somatic mutations were recurrently identified to correlate with the impact on the immune response by affecting immune-related cells in the TME (18). According to our model, CD8 T cells, macrophage cells, and helper T cells were the main force during the response to ICIs. McGrail et al. identified that CD8 T cell infiltration exhibited a significantly positive correlation with TNB in LUAD and an opposite pattern in LUSC (19). The MT group showed increased TNB, increased immune infiltrating cells, and upregulated checkpoint molecules, which suggested noticeable immune-supportive features (25). A high level of M1 macrophage cells has been reported to be positively correlated with TMB-H, mainly because M1 macrophages can provide an anti-tumor environment by fostering an inflammation response against tumor-activating CD8 T cells (5). Follicular helper T cells were identified as positively correlated with increased B cell activation capacity, which could help include local antibodies in predicting tumor response to immunotherapy (26). Single-cell RNA sequencing analysis revealed the expression distribution in different cell types, and most of the model genes were enriched in T cells, macrophage cells, and B cells. Thus, the 18 genes may affect the TME by recruiting immune-activated cells and neoantigens to respond ICIs well in NSCLC. Pathway analysis showed significant enrichment of transcription factors, cell cycle pathway, HR pathway, MMR pathway, and DNA replication pathway in the MT group. Some previous studies confirmed that deficiencies in the DDR pathway are closely related to immunotherapy outcomes (27). The cell cycle pathway is positively associated with increased TMB and response to PD-L1 blockade (28). According to our study, gene alterations in tumor cells could affect the heterogeneous expression of immune cells and interactions in the TME, consequently leading to different outcomes for ICI treatments.

Although our model shows an advantage over some reported predictive biomarkers, algorithms and sample size differences may have affected the analyses. However, it still opens a new option of combining the model with previously identified biomarkers to develop a better ICI therapeutic efficacy predictor.

With the rapid development of next-generation sequencing (NGS) technology and improved bioinformatic analysis methods, tumor treatment has become a more distinct era, which aims to help more patients survive longer with fewer adverse effects. Fortunately, our model could predict the long-term survival of patients with NSCLC treated with immunotherapy. The number of mutant genes within the model is the only evaluation criterion that could be easily applied in clinical and commercial detection. The results have strengthened our confidence that our model could select patients with NSCLC who could benefit from immunotherapy.

## 4 Material and methods

### 4.1 Data source

The training cohort included 344 patients with NSCLC treated with ICIs (Immunotherapy, MSKCC, Nat Genet 2019) (9). Validation cohort 1 was integrated from three public cohorts sequenced by the WES, including 56 patients with NSCLC treated with anti-PD-(L)1 therapy (15), 75 patients with NSCLC treated with PD-1 plus CTLA-4 blockade (LUAD only) (16), and 69 patients with NSCLC treated with anti-PD-(L)1 monotherapy at Sun Yat-sen University Cancer Center (SYSUCC) (17). Validation cohort 2 was a pan-cancer cohort including 1181 patients treated with anti-PD-(L)1 therapy (From Samstein cohort, 350 patients with NSCLC, and 130 patients with unknown cancer were excluded) (9). Validation cohort 3 was also a pan-cancer cohort including 193 patients treated with anti-PD-(L)1 therapy (15) (From Miao cohort, 56 patients with NSCLC were excluded). Both training and validation cohorts were selected based on the following criteria: (i) patients with no mutation information were excluded; (ii) synonymous mutation, copy number variation, and fusion genes were excluded; (iii) genes were mutated in at least three samples. In addition, data from non-ICI treatment TCGA NSCLC cohorts were used for further exploration, including RNA-seq data downloaded from UCSC Xena (University of California Santa Cruz) (https://xenabrowser.net/datapages/), immune subtype data along with survival data acquired from Thorsson et al. (18), and mutation data obtained from Ellrott et al. (29). In addition, six single-cell RNA sequencing data of LUAD patients from Bischoff, P., et al. (30) were included to reveal the gene expression features in different cell types (30). In addition, a retrospective southwest hospital clinical (SHC) cohort, with 82 lung cancer patients, was utilized to analyze the correlation between the predictive model and TMB. Of these, 77 were NSCLC, and the remaining were primary lung cancer. Survival data could not be acquired because of the loss of follow-up after surgery. All the samples were collected in the Southwest Hospital, and multiple gene panel target sequencing was conducted. The detailed clinical characteristics of patients in the training cohort, validation cohort 1-3, TCGA cohort, and SHC cohort are summarized in **Supplementary Tables 2–7**. The detailed mutations data of SHC cohort are listed in **Supplementary Table 8**.

### 4.2 Next-generation sequencing and mutation analysis

Genomic profiling was performed on tumor tissues and matched peripheral blood samples. First, we used the Maxwell RSC FFPE Plus DNA Kit (Promega, Cat no.AS1720) to extract DNA from tumor specimens and blood, respectively. Then, 100ng gDNA was sheared to target 200 bp fragment sizes with a Covaris E210 system (Covaris, Inc.). Next-generation sequencing of gDNA was performed, in which KAPA HyperPrep Kit (Roche, 07962312001) and Agilent SureSelect XT kit (Agilent, G9702C) were used to construct the NGS library. The prepared library was quantified using the Qubit 3.0 Fluorometer (Life Technologies, Inc.), and quality and fragment size were measured with an Agilent 2100 Bioanalyzer (Agilent Technologies, Inc.). Samples underwent paired-end sequencing on an Illumina NovaSeq 6000 platform (Illumina Inc) with a 150-bp read length.

### 4.3 Data processing

Burrows-Wheeler Aligner (BWA version 0.7.11) alignment algorithm was used to align the human reference genome (UCSC hg19). Next, Genome Analysis Toolkit (GATK, version 3.6) (31) module IndelRealigner and VarScan software were used to call somatic mutations (31, 32), and ANNOVAR annotated all variants. The following filtering criteria were applied to the mutation candidates to identify SNVs and Indels: (a) variants within intron were deleted; (b) mutations reported in more than 1% of the population in the 1000 Genomes Project (1000gAUG_2015ALL); (c) Mutations were then filtered against common single nucleotide polymorphisms (SNPs) found in dbSNP (http://www.ncbi.nlm.nih.gov/SNP); (d) synonymous variants were excluded; (e) variants with less than 50 supporting reads were removed.

### 4.4 Genomic biomarkers analysis

Tumor tissue MSI status was determined by MSIsensor software (33) in the form of an MSI score. TMB was determined by using the number of all nonsynonymous mutations and indels per megabase of the genome examined. The cut-off value for TMB-high and TMB-low was defined as 10 mutations/Mb.

### 4.5 Feature importance ranking using random forest algorithm

A random forest (RF) (33) classifier was applied to the training cohort to explore the most critical mutant genes associated with the overall survival of ICI-treated NSCLC patients. We used the variable importance measure of the RF algorithm to rank gene features. Scikit-learn, a Python machine-learning library, was used to find the feature gene. Then, the sequential backward search (SBS) method was used to remove several features with minor importance scores from the feature set during each iteration. The classification accuracy was calculated after each round of screening to find the best subset of features in time.

In the training process, we adopted 10-fold cross-validation and considered the average classification accuracy rate as a measure to ensure the stability of the experimental results. At the beginning of filtering, the step size was relatively large due to many features. When there were 400 features, the step size was 100. Gradually, as the number of features decreased, the step size dropped to 50 and 20. Lastly, when there were only 50 genes left, the step size was reduced to 1 until the end of the experiment. Finally, a feature gene set with the least number of variables and the highest classification accuracy rate was obtained to develop a predictive model.

### 4.6 Mutation-based model development and validation

Univariate and multivariate Cox regression methods were used to compare clinical factors and predictive models. Based on the selected gene features, patients were divided into the mutation-type (MT) group (mutant gene ≥ 2) and wild-type (WT) group (mutant gene < 2). The same grouping criteria and analysis methods were applied for the validation cohorts.

### 4.7 Immunogenomic feature evaluation

Immunogenomic features were obtained from a previous pan-cancer immune landscape project performed by Thorsson et al. (18). In brief, TNB (tumor neoantigen burden) was defined as a critical target of anti-tumor immunity and calculated by the NetMHCpan algorithm (34). HRD score was used to evaluate the deficit by summation of loss of heterozygosity (LOH), large-scale transitions (LST), and genomic instability scores (GIS) (35). The relative abundance of 22 immune cell types was estimated by the CIBERSORT algorithm (36).

### 4.8 Single-cell RNA sequencing analysis

Six single-cell RNA sequencing data of LUAD from the Bischoff cohort were enrolled to analyze the expression features of 18 model genes in the TME. The scRNAseq expression matrix was processed with R package “Seurat”. UMAP reduction was used for cluster visualization, and the “SingleR” package was used for cluster annotation. “FeaturePlot” and “VlnPlot” were used to visualize gene expression.

### 4.9 ssGSEA and differential analysis

The single-sample enrichment score of Kyoto Encyclopedia of Genes and Genomes (KEGG) pathways, IFN-γ pathway markers (20), and m6A regulators (37) were calculated by single-sample gene set enrichment analysis (ssGSEA) algorithm using the R package ‘GSVA’ (38). Pathway differential analysis was conducted using R package ‘limma’ with a Benjamini-Hochberg (BH) corrected p-value threshold of 0.01 and visualized by R package ‘pheatmap’ (39).

### 4.10 APOBEC mutational signature score calculation

APOBEC enrichment score was calculated using the R package ‘maftools’ and survival difference was compared with the log-rank test.

### 4.11 Statistical analysis

Survival curves were compared using the Kaplan-Meier (KM) method. Model receiver operating characteristic (ROC) curves were analyzed, and the predictive performance was evaluated using the area under the ROC curve (AUC) value. Statistical difference between the two groups was analyzed using the Wilcoxon test, and a *P* value < 0.05 was considered statistically significant. R software (4.1.2) was applied to carry out all statistical analyses.

## Data availability statement

The datasets presented in this study can be found in online repositories. The names of the repository/repositories and accession number(s) can be found in the article/**Supplementary Material**.

## Ethics statement

All the patients in the SHC cohort were retrospectively collected in the Southwest Hospital and provided written informed consent. This study was approved by the ethics committee of Southwest Hospital (Number KY2022200).

## Author contributions

Conception and design of the study: PH, JL, QX, GH, WW. Acquisition of data: HM. Data analysis: PH, JL. Visualization: QX, HM. Review & editing: BN. Original draft: PH, JL, QX. Supervision of the study: GH, WW. All authors contributed to the article and approved the submitted version.
