# Supplementary material for: Development and validation of a mutation-based model to predict immunotherapeutic efficacy in NSCLC
Source: Front Oncol. 2023 Feb 24;13:1089179. doi: 10.3389/fonc.2023.1089179 (PMC9998990; doi:10.3389/fonc.2023.1089179)
Supplement: Supplementary file 2 [file DataSheet_2.docx]

Supplementary Material

## Supplementary Figures

**
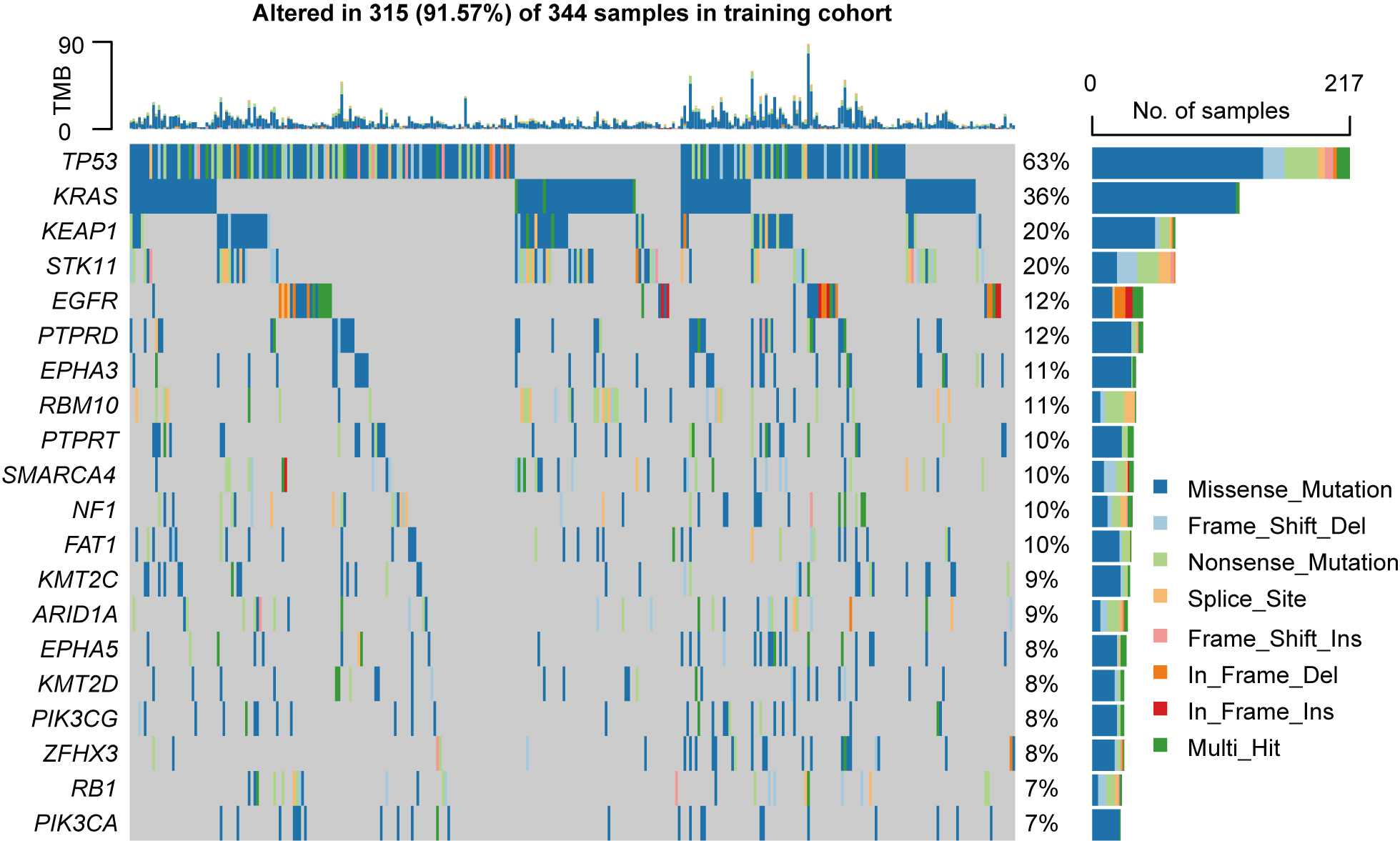
**

**Supplementary Figure 1.** **Mutational landscape of NSCLC patients in the training cohort.**
The left and right panel indicates mutational genes and their mutation frequency separately; the upper panel shows mutational prevalence concerning different mutation types, the middle panel depicts the gene mutation landscape across analyzed cases with different mutation types color-coded differently.


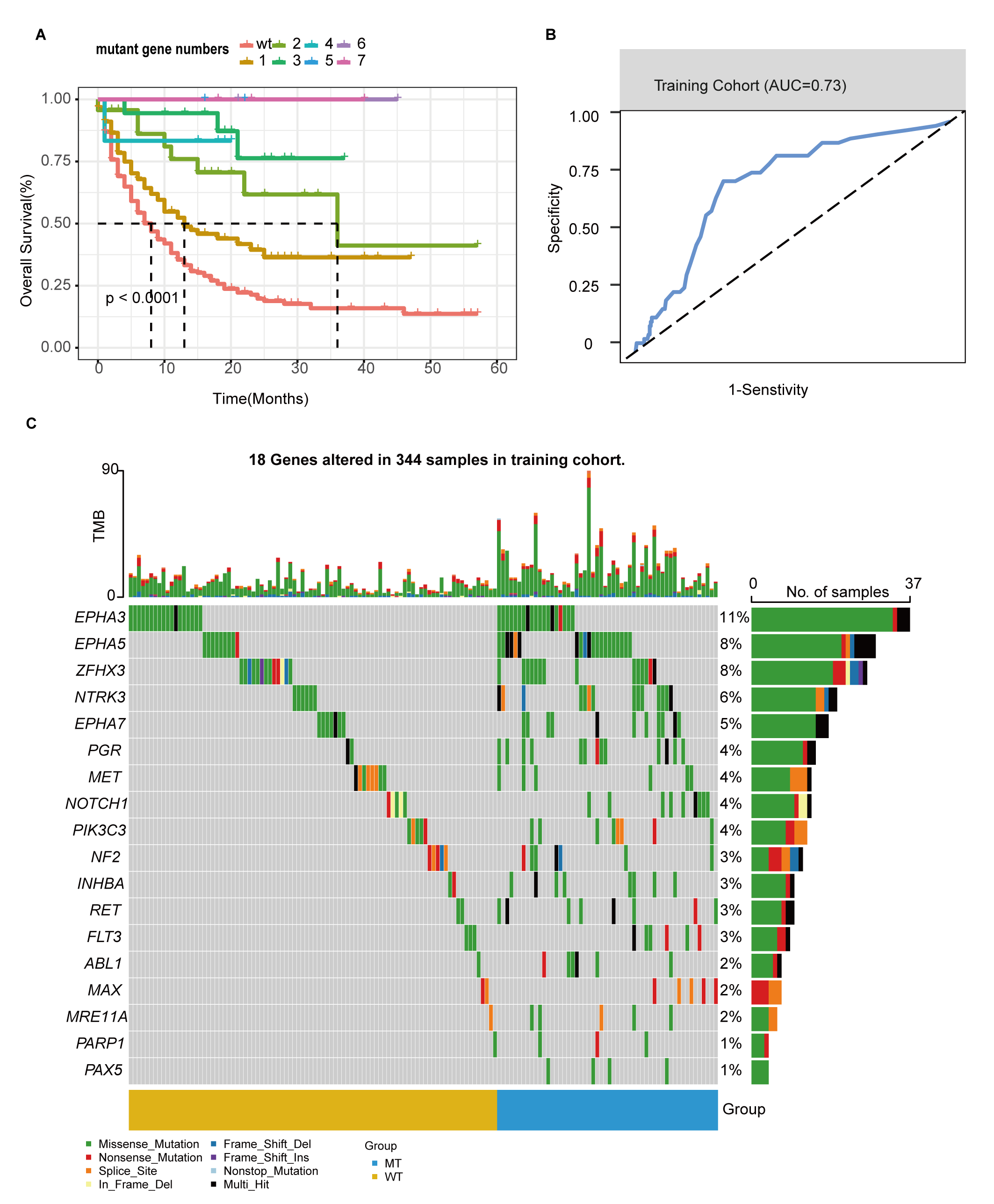


**Supplementary Figure 2.** **Predicting the immunotherapeutic efficiency using mutation-based model in the training cohort. (A)** Survival analysis across different subgroups according to the mutant gene number of 18 genes in the training cohort. **(B)** The AUC analysis for mutation-based model in the training cohort. AUC, Area under the ROC curve. **(C)** Mutation landscape of 18 genes in the training cohort.
The left and right panel indicates mutational genes and their mutation frequency separately; the upper panel shows mutational prevalence concerning different mutation types, the middle panel depicts the gene mutation landscape across analyzed cases with different mutation types color-coded differently, and the bottom panel displays group features based on the mutation-based model, including MT and WT group.


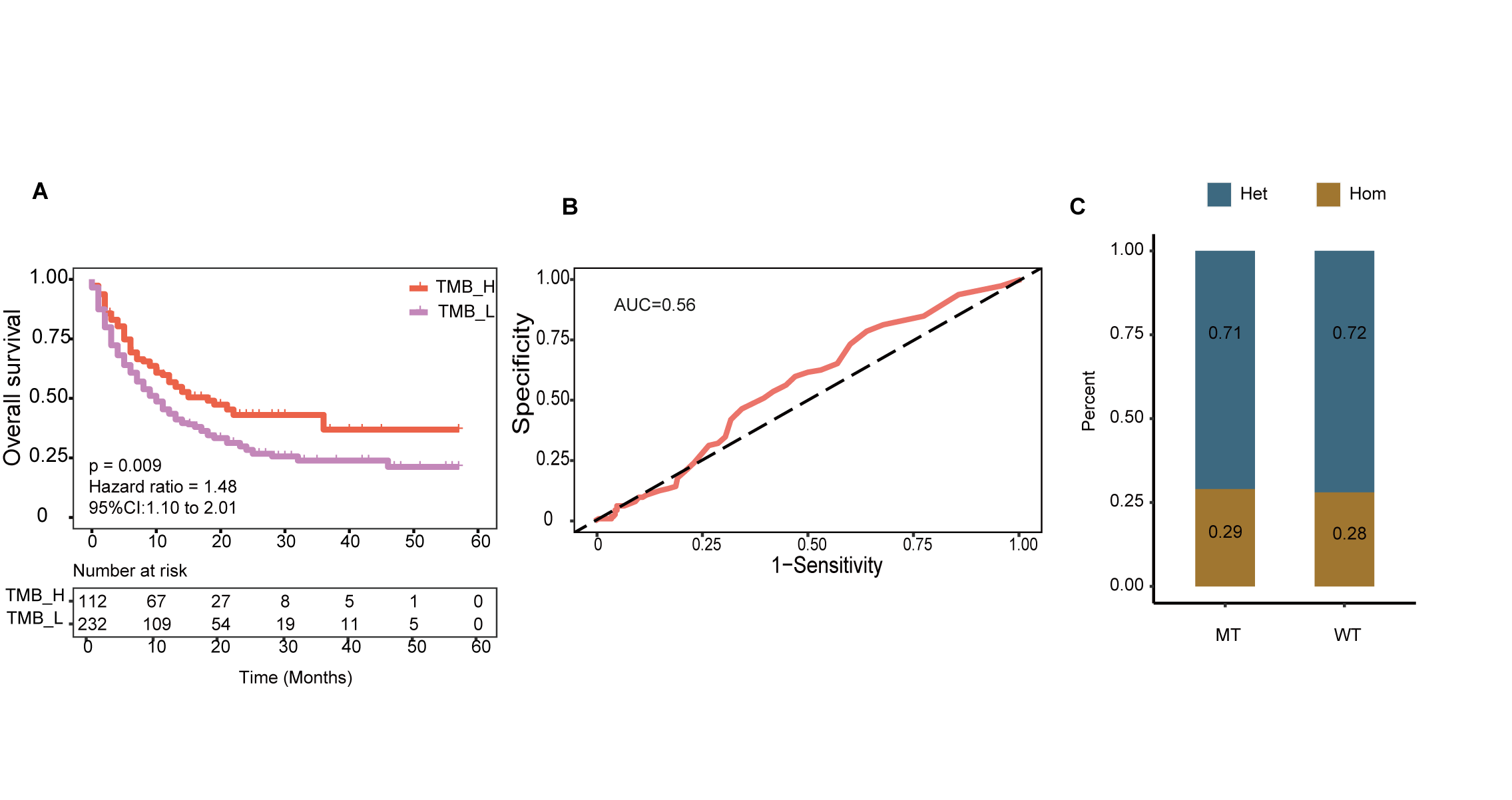


**Supplementary Figure 3.** **TMB level predicting ICIs response and HLA variability analysis. (A)** Kaplan-Meier survival analysis for OS between TMB-H and TMB-L group in the training set. **(B)** The AUC analysis for TMB in the training cohort. AUC, Area under the ROC curve. **(C)** HLA variability analysis in SHC cohort. Het: heterozygous; Hom: homozygous.


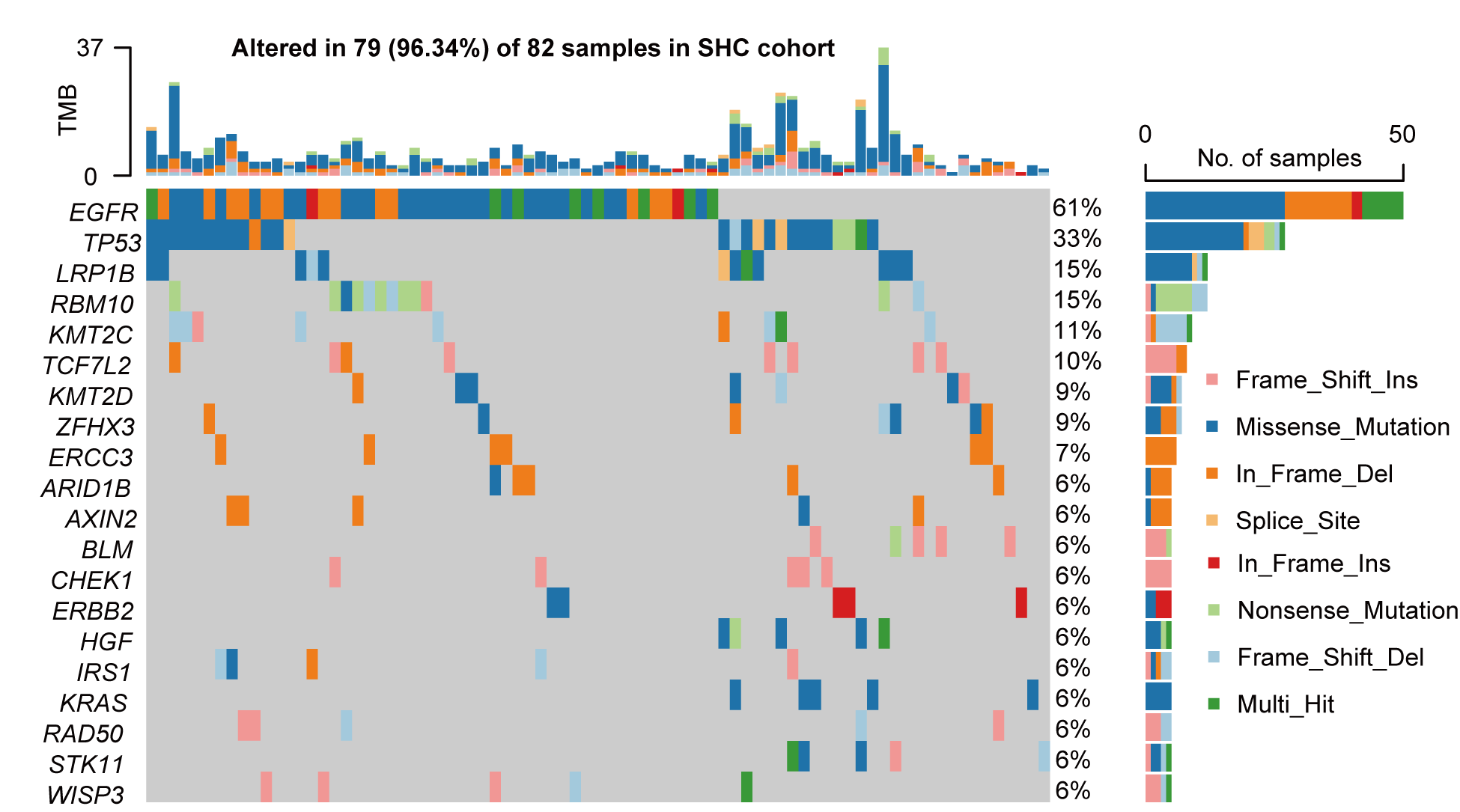


**Supplementary Figure 4.** **Mutational landscape of NSCLC patients in the SHC cohort.**
The left and right panel indicates mutational genes and their mutation frequency separately; the upper panel shows mutational prevalence concerning different mutation types, the middle panel depicts the gene mutation landscape across analyzed cases with different mutation types color-coded differently.


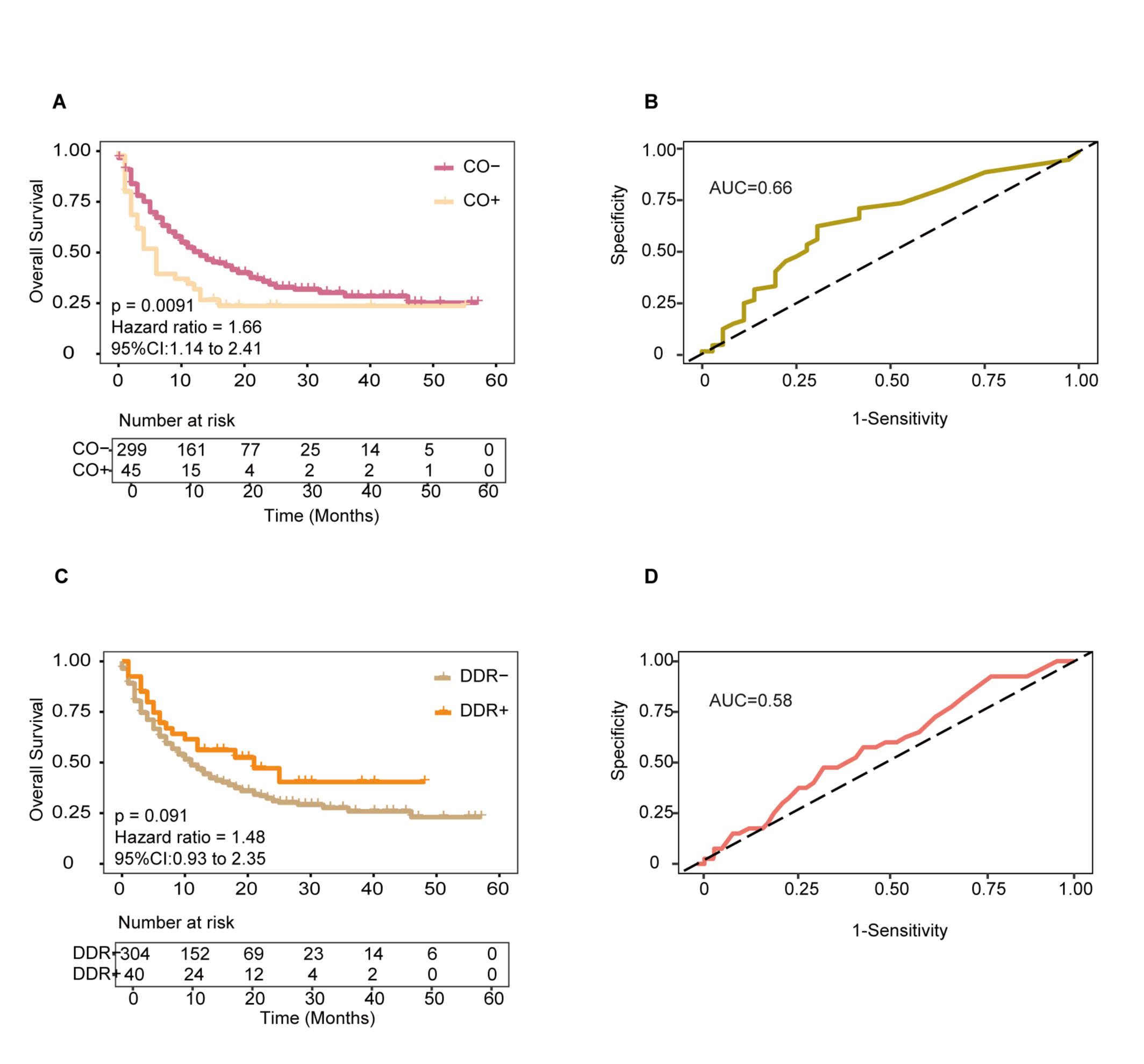


**Supplementary Figure 5.** **Predictive performance of previously reported biomarkers.** **(A)** Kaplan-Meier survival analysis for OS between patients with *KEAP1-*driven co-mutation (CO+) and patients without *KEAP1-*driven co-mutation (CO-) in the training set. **(B)** The AUC analysis for *KEAP1*-driven co-mutations in the training set. **(C)** Kaplan-Meier survival analysis for OS between patients with more than or equal to one of five DDR gene mutations (DDR+) and patients with none of five DDR gene mutations (DDR-) in the training set. **(D)** The AUC analysis for DDR pathway genes in the training set.


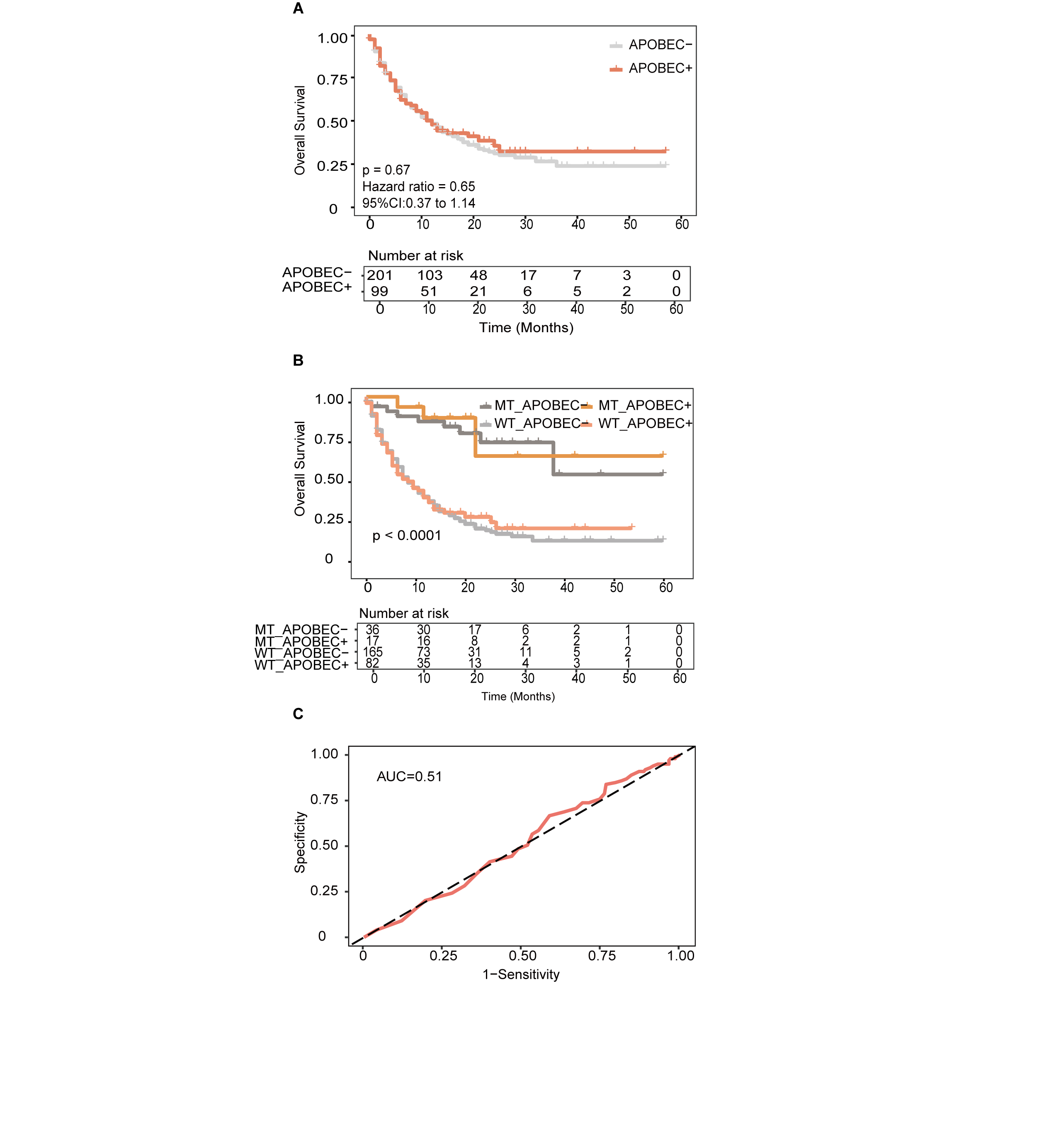


**Supplementary Figure 6.** **Predictive performance of APOBEC signature.** **(A)** Kaplan-Meier survival analysis for OS between patients with more than or equal to one APOBEC-related gene mutation (APOBEC+) and patients without APOBEC-related gene mutations (APOBEC-) in the training set. **(B)** Comparing the predicting ability of APOBEC gene signature with the mutation-based model in ICIs treatment outcomes. **(C)** The AUC analysis for APOBEC gene signature in the training set. AUC, Area under the ROC curve.


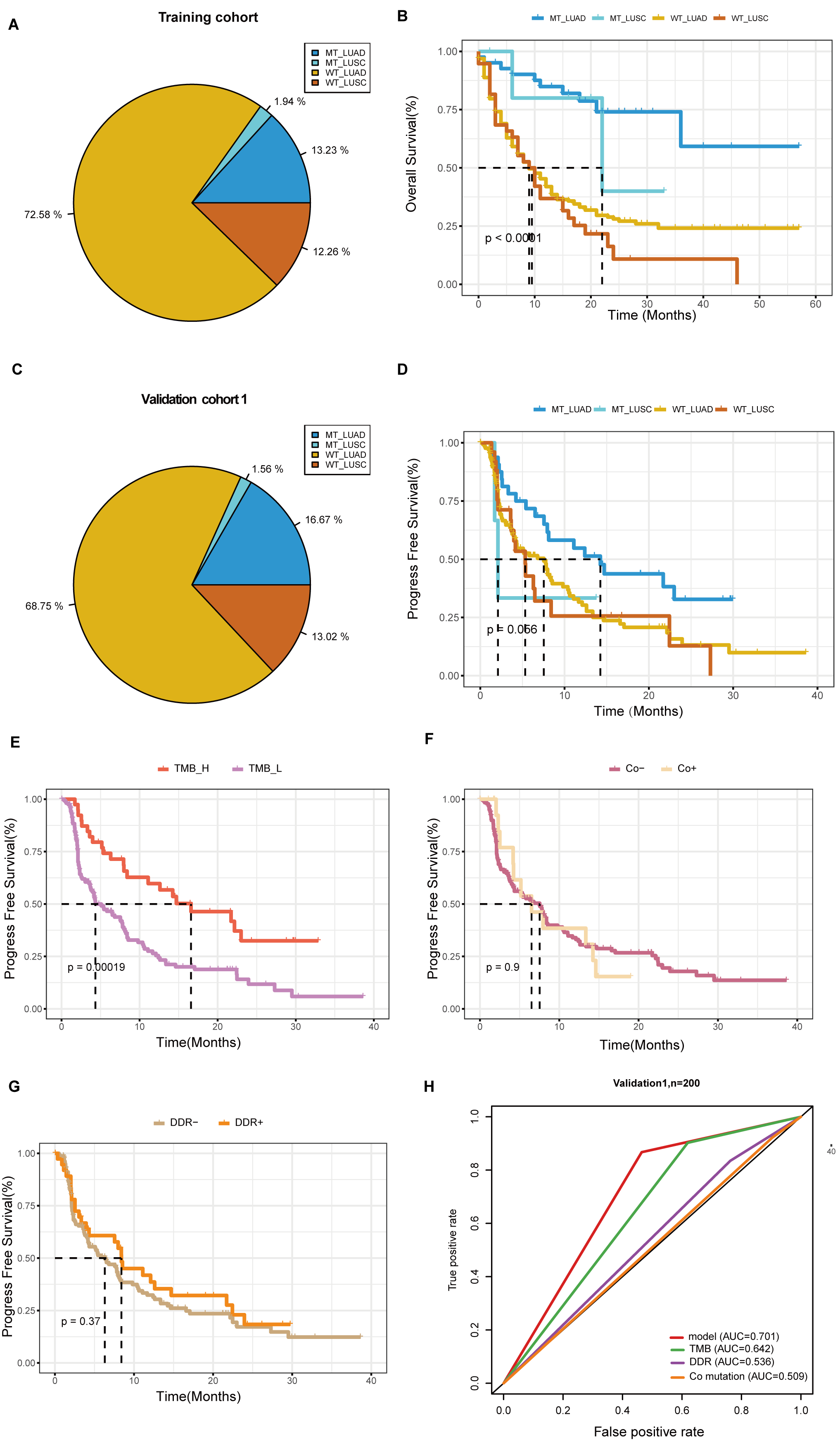


**Supplementary Figure 7.** Predicting performance analysis within two NSCLC subtypes and performance comparison with other biomarkers. **(A)** The pie chart shows the proportion four subgroups in the training cohort. **(B)** Kaplan-Meier survival analysis for OS between four subgroups in the training cohort. **(C)** The pie chart shows the proportion four subgroups in the validation cohort 1. **(D)** Kaplan-Meier survival analysis for PFS between four subgroups in the validation cohort 1. **(E)** Kaplan-Meier survival analysis for PFS between TMB-H and TMB-L group in the validation cohort 1. **(F)** Kaplan-Meier survival analysis for PFS between patients with *KEAP1-*driven co-mutation (CO+) and patients without *KEAP1-*driven co-mutation (CO-) in the validation cohort 1. **(G)** Kaplan-Meier survival analysis for PFS between patients with more than or equal to one of five DDR gene mutations (DDR+) and patients with none of five DDR gene mutations (DDR-) in the training set.**(H)** The AUC analysis for the TMB, *KEAP1*-driven co-mutation, and DDR in the validation cohort 1. ROC, receiver operating characteristic; AUC, Area under the ROC curve; The four subgroups including MT_LUAD, MT_LULSC, WT_LUAD, and WT_LUSC.


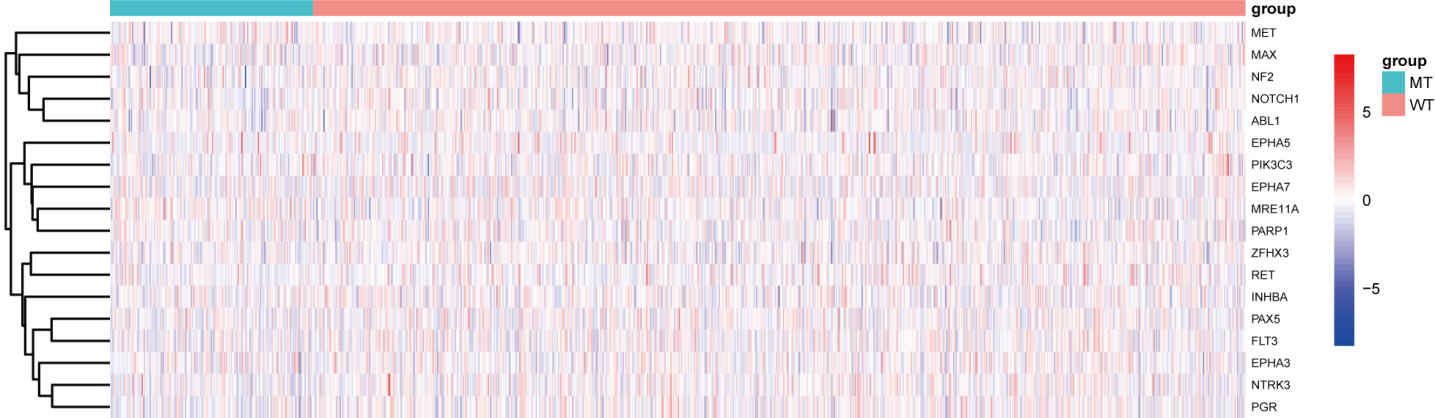


**Supplementary Figure 8.** The heatmap of the RNA expression level of 18 genes in TCGA-NSCLC.


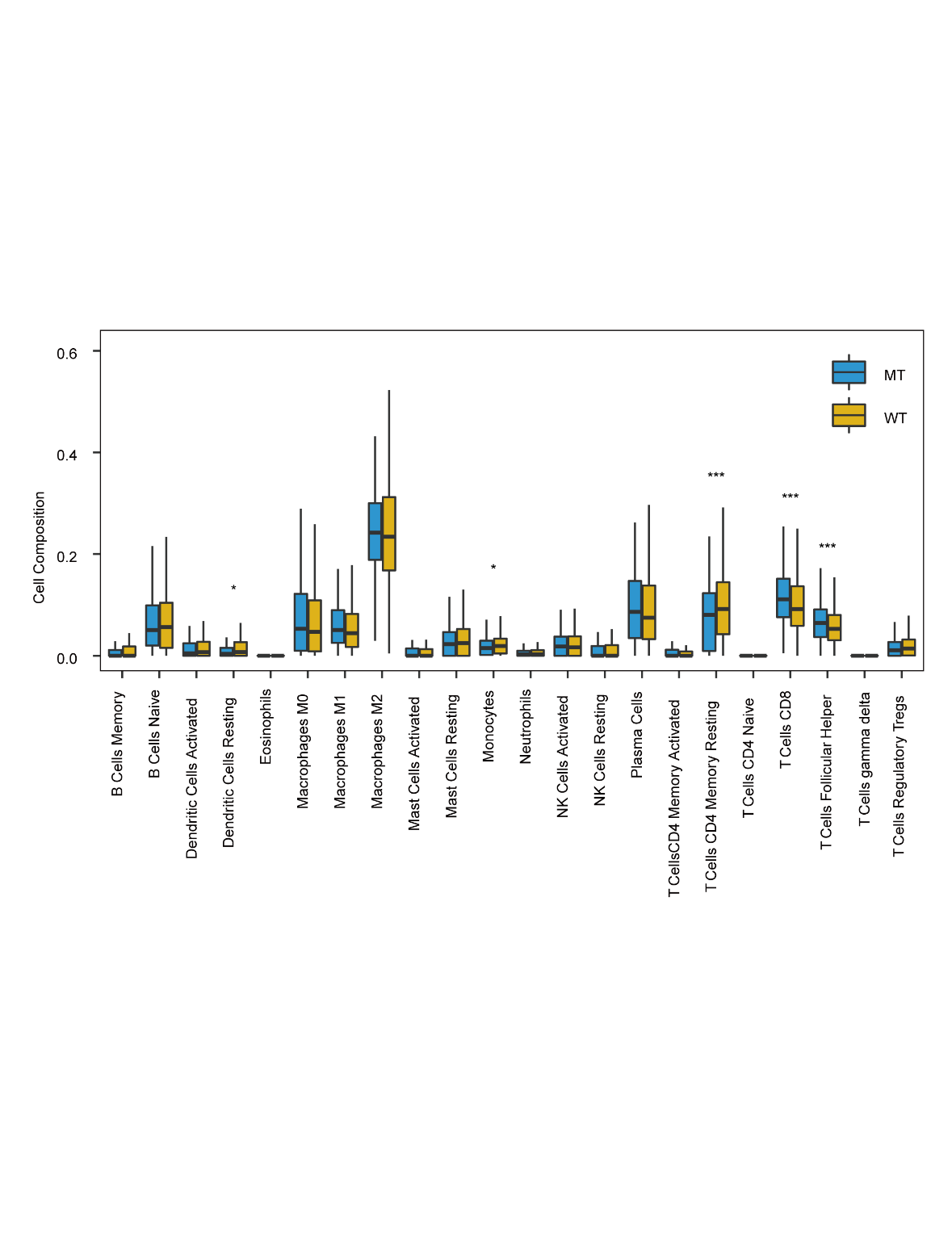


**Supplementary Figure 9.** **Immune infiltration analysis of NSCLC between WT and MT groups.** WT group: wild-type gene; MT group: mutation-type gene group.


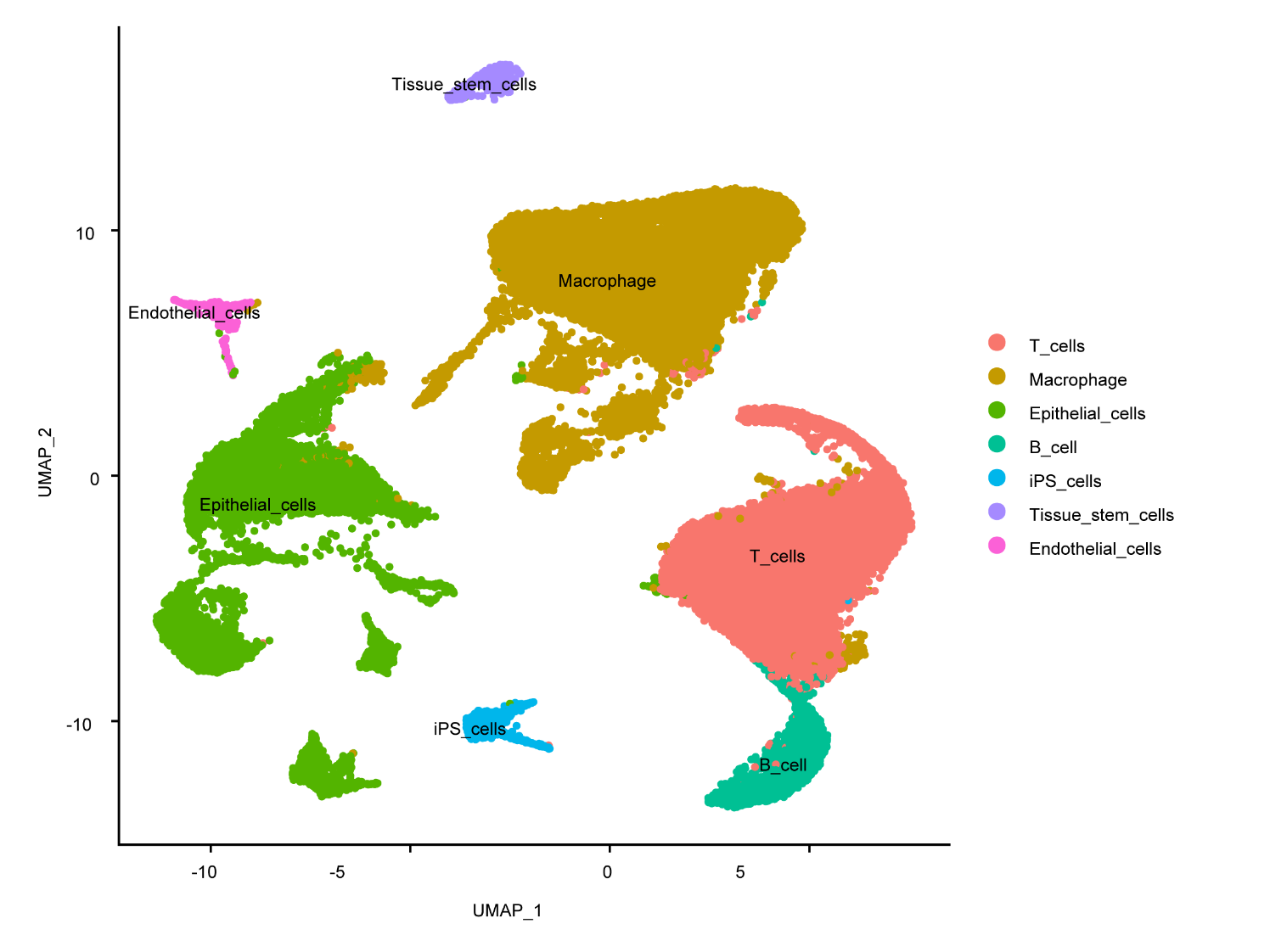


**Supplementary Figure 10.** **UMAP dimensionality reduction was used to show the distribution and dissimilarity of the seven cell types in LUAD.**

**
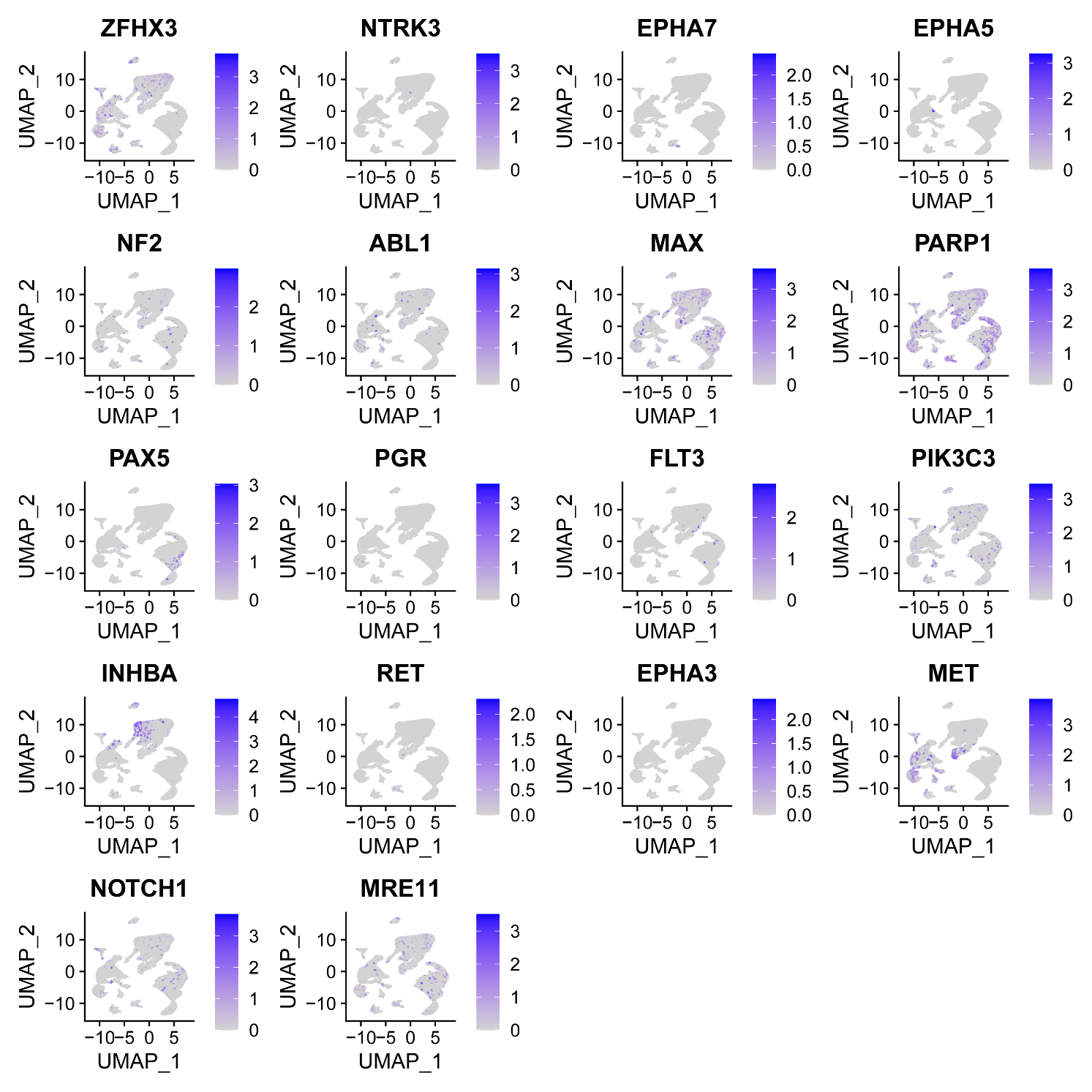
**

**Supplementary Figure 11.** **Distribution of 18 genes in seven cell types in LUAD.**


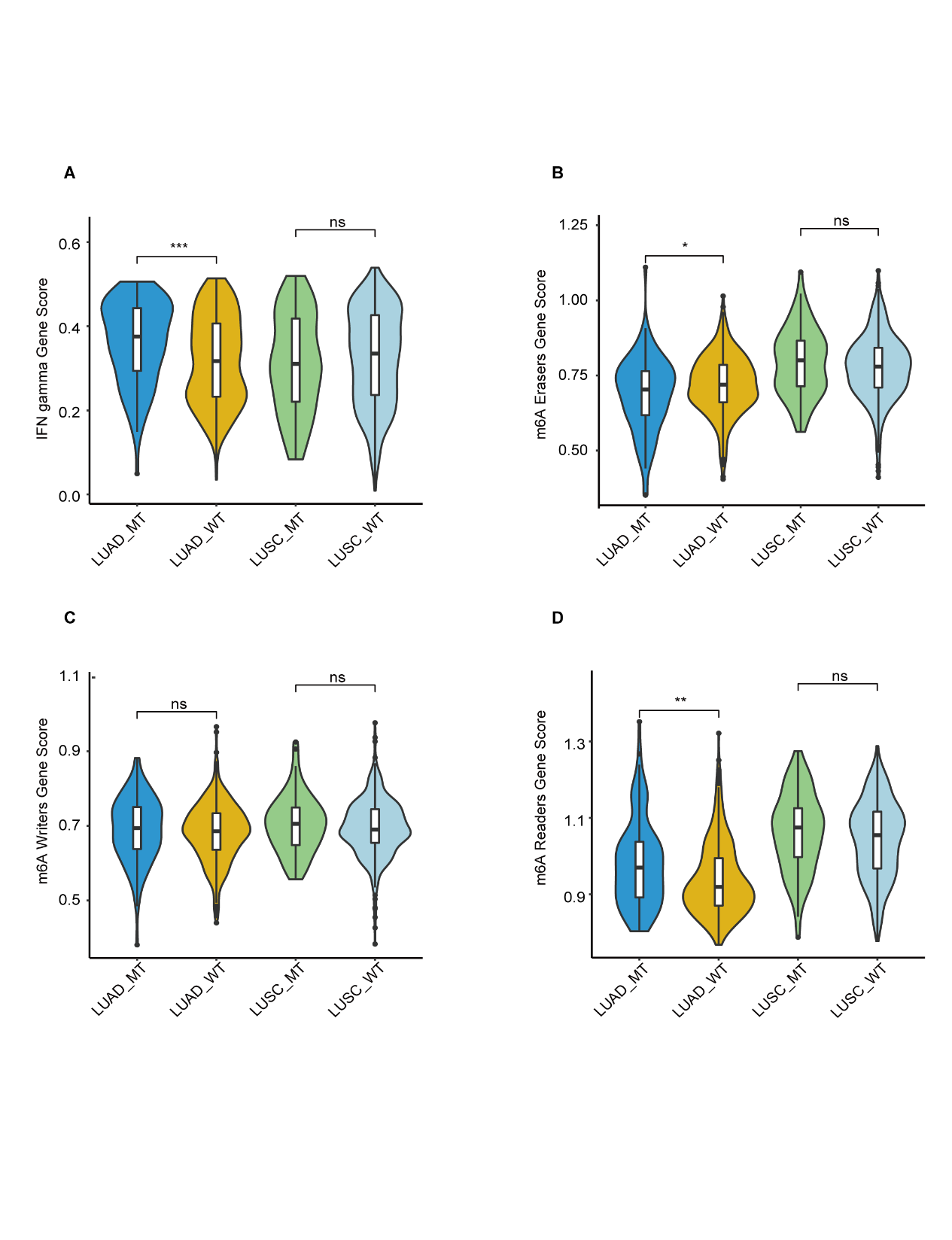


**Supplementary Figure 12.** **Immune-related features analysis of NSCLC between WT and MT groups. (A)** IFN-γ gene signature score. **(B)** erasers, **(C)** writers, and **(D)** readers of m^6^A genes signature score among four groups of LUAD_MT, LUAD_WT, LUSC_MT, and LUSC_WT. **P*≤0.05; ***P*≤0.01; ****P*≤0.001; *****P*≤0.001; ns: not significant. LUAD: lung adenocarcinoma; LUSC: lung squamous cell carcinoma.


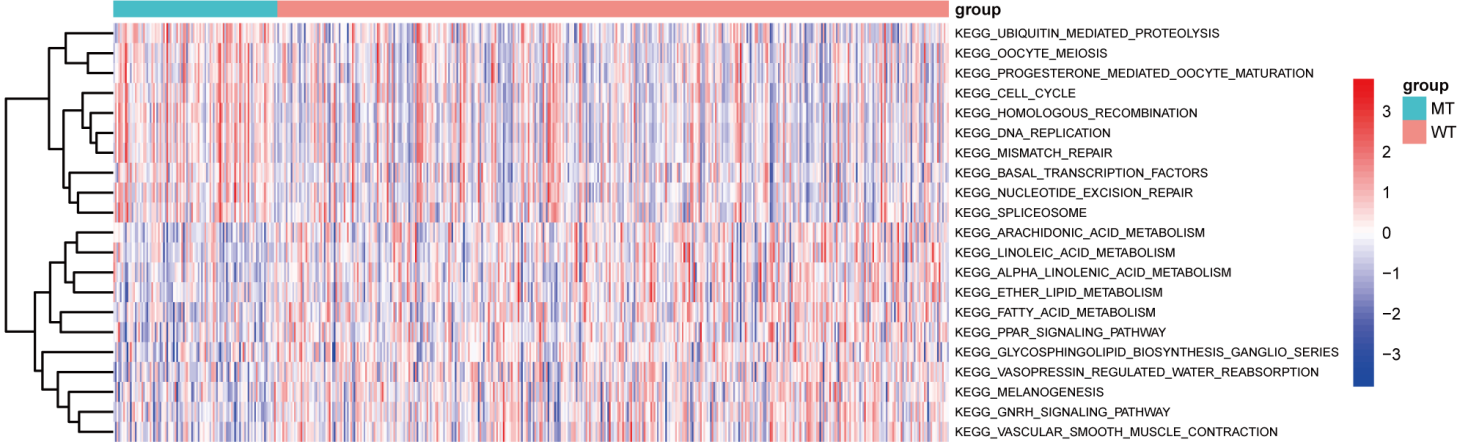


**Supplementary Figure 13.** **The heatmap of KEGG pathway enrichment.**
